# Leveraging electrochemical double layer structure to rationally control electrolysis

**DOI:** 10.1093/nsr/nwae299

**Published:** 2024-08-24

**Authors:** Gong Zhang, Marcel Schreier

**Affiliations:** Department of Chemical and Biological Engineering, University of Wisconsin-Madison, USA; Department of Chemical and Biological Engineering, University of Wisconsin-Madison, USA; Department of Chemistry, University of Wisconsin-Madison, USA

## Abstract

This perspective delves into the electrochemical microenvironment, uncovering entropic effects in CO_2_ reduction, revealing neutral molecule electrosorption under polarization, highlighting challenges in the classical double layer model, and proposing research approaches for future interface studies.

The interface created when contacting an electrode with an electrolyte is remarkably diverse, characterized by uneven distributions of charge density and the arrangement of ionic species and solvent molecules. This region, which governs the function of all electrochemical devices, is commonly termed the electrical double layer (EDL), a concept originated by Helmholtz in 1853 [1]. To date, the EDL structure is often described simplistically. For example, some models such as the Gouy–Chapman–Stern (GCS) model describe it as a collection of distinct layers of ions (inner Helmholtz plane, outer Helmholtz plane, and diffuse layer, Fig. 1a). Such simple descriptions fall short of depicting the complex reality characterizing electrochemical interfaces at the molecular level, thereby hampering our understanding of electrochemical processes and their rational control. In this Perspective, we highlight examples of recent research from our group and others that show the importance of a molecular-level view of chemical interactions at electrochemical interfaces. These approaches open novel avenues for leveraging the impact of interfacial organization on electrocatalytic reaction rates and for controlling the potential-dependent adsorption of neutral species. We anticipate that these concepts will benefit research related to energy storage and sustainable chemical synthesis.

**Figure 1.**
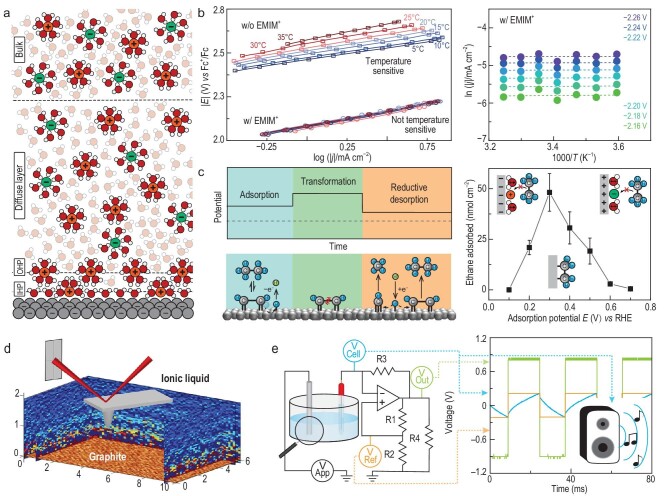
The classical EDL model and recent examples of research studying and leveraging interfacial structure. (a) Schematic of the classical GCS model. Not drawn to scale. IHP: inner Helmholtz plane. OHP: outer Helmholtz plane. (b) CO_2_ reduction Tafel slopes (lines correspond to data, dots correspond to fits, left) and corresponding Arrhenius-like plots on an Ag electrode in the presence of 50 mM EMIM^+^ (right). Adapted with permission from Ref. [4]. Copyright 2023 American Chemical Society. (c) Schematic of the independently voltage-controlled steps allowing for room-temperature ethane fragmentation reaction (left). Adsorbate structures are hypothetical and alternative structures are possible. Adapted with permission from Ref. [8]. Copyright 2023 American Chemical Society. Potential-dependent adsorption of ethane on a Pt electrode in 1 M H_2_SO_4_ (right). Adapted with permission from Ref. [7]. Copyright 2024 American Chemical Society. (d) A 3D-AFM image of one area of the electrode–electrolyte interface. Adapted with permission from Ref. [9]. Copyright 2020 American Chemical Society. (e) Visual depiction of the connection between EDL formed between the working electrode and the electrolyte (magnifying glass) and the relaxation oscillator circuit. Adapted with permission from Ref. [10]. Copyright 2024 American Chemical Society.

**Kinetic role of potential-dependent pre-exponential factor.** Basic electron transfer models, including the Tafel, Butler–Volmer, and simple Marcus models, suggest that the applied potential controls reaction rates by altering the Gibbs free energy of activation (Δ*G*^‡^) [2]. Given that Δ*G*^‡^ depends on both the enthalpy change of activation (Δ*H*^‡^) and entropy change of activation (Δ*S*^‡^), there is ongoing interest in whether changes in electrochemical reaction rates are driven more by Δ*S*^‡^ or Δ*H*^‡^. Based on the work of Conway *et al*., which separates the transfer coefficient into entropic and enthalpic parts [3], it is suggested that Δ*S*^‡^ could play a significant role in regulating electrochemical reaction rates. Recently, our measurements have provided some experimental evidence that the rate of electrochemical reactions could indeed be influenced by effects related to the entropy of activation (Δ*S*^‡^). We have studied the temperature-dependence of the electrochemical CO_2_ reduction reaction (CO_2_RR) on silver electrodes in an acetonitrile electrolyte containing imidazolium cations [4]. In the presence of 1-ethyl-3-methylimidazolium (EMIM^+^), CO_2_RR appears to be independent of temperature, with the apparent activation energy (*E*_A, app_) approaching zero, while the potential still affects the reaction rate. We propose that these observations suggest that under these conditions the reaction rate is influenced by effects related to the entropy of activation, which may be related to the ordering of ions at the interface (Fig. 1b). As such, in this system, the reaction may at least partially be controlled by the potential-driven interfacial organization. Indeed, precedent exists for rate control through entropic double layer effects [5,6]. For example, it has been suggested that for the hydrogen evolution reaction (HER), adding metal cations to the electrolyte leads to the ordering of H_2_O at the interface (an entropy decreasing process), thus increasing the rate of HER [5]. These observations suggest that to understand the rate of electrochemical reactions, interfacial organization (i.e. the arrangement of anions, cations, or solvent molecules near the electrode surface under the influence of an electric field) cannot be ignored. We anticipate that exploiting the relationship between potential-dependent organization of electrolyte components and reaction kinetics can significantly enhance the engineering of efficient energy storage systems and electrosynthesis processes. Furthermore, we expect that the correlation between interfacial organization and electrochemical reaction rates could be validated in the future using spectroscopic or imaging techniques.

**Understanding the adsorption of neutral species within the EDL paves the way to novel electrocatalytic reactions.** The organization of electrolyte components at the electrochemical interface is also critical to the modulation of adsorption and desorption processes on electrode surfaces using the applied potential. For example, a comparably poorly appreciated feature of electrochemical interfaces is that the adsorption of neutral compounds to electrode surfaces is potential-dependent. When the electrode potential is close to the potential of zero charge (PZC), where the actual surface excess charge density becomes zero, the interaction between the polar solvent or ions and the electrode surface is minimized, allowing for the adsorption of neutral molecules. However, when the potential deviates from the PZC, the interface recruits solvents and ions, displacing neutral molecules and hindering their adsorption [7]. Thus, modulating the electrical field provides a fast method for tuning the coverage of neutral compounds on the electrode surface. Our group has leveraged this fact to realize the potential-controlled adsorption and subsequent electrochemical cracking of ethane to methane at room temperature. When adsorbing ethane to a platinum electrocatalyst surface, we found that adsorption was maximized at 0.3 V *vs* the reversible hydrogen electrode (RHE) and decreased sharply at more positive and negative potentials. By applying oxidative potentials after ethane adsorption, we subsequently promoted the scission of surface-bound ethane to C_1_ fragments at the catalyst surface (Fig. 1c). Leveraging the potential-dependence of the adsorption of neutral species then allowed us to apply a more reductive potential of 0.05 V *vs* RHE to desorb the C_1_ fragments in the form of methane, thus accomplishing the room-temperature fragmentation of ethane to methane which requires 300–400°C to occur on Pt under thermal conditions [8]. These measurements were made possible by using electrochemical mass spectrometry (EC-MS), which allows for monolayer-sensitive studies of electrochemical transformations. We expect that the rational real-time tuning of electrochemical interfaces at each step of an electrocatalytic reaction will allow for the customization of individual reaction steps.

**Seeing and hearing the structure of the electrochemical double layer.** Fully leveraging the complex nature of electrochemical interfaces as described in the examples highlighted above requires methodologies to gain insight into the structure of the EDL. Encouraging developments have used electrochemical three-dimensional atomic force microscopy (EC-3D-AFM, Fig. 1d) to allow the observation of the organization of EDL components at the molecular level [9]. These techniques emphasize the potential-dependent reorganization of solvent molecules, as well as the periodic ordering of electrolyte components close to the electrode surface, all of which are not adequately represented by simplistic models such as the GCS model. To make these complex dynamics more intuitive to understand, our group has recently demonstrated a method for representing the behavior of the EDL through sound [10]. By employing the EDL as a variable element in a relaxation oscillator circuit, an auditory waveform is generated (for detailed methods and representative audios, please refer to Ref. [10]), which, once connected to a speaker, audibly represents the rearrangement of the components constituting the EDL (Fig. 1e). Changes in applied working electrode potential, electrode material, electrolyte ion concentration and identity alter the EDL capacitance. In our oscillator circuit, these changes in capacitance can produce frequency-variable output waveforms, offering intuitive insights into the complex dynamics at electrochemical interfaces.

We hope that the research discussed herein will draw attention to the fascinating dynamics taking place upon polarization of electrochemical interfaces. We expect that exploring the thus far hidden properties of the EDL and gaining an insight of how they control electrochemical reactions will unlock further progress in energy storage and in the electrosynthesis of fine and bulk chemicals.
